# First Detection of *Alphacoronavirus* in Bats from the World’s Largest Wetland, the Pantanal, Brazil

**DOI:** 10.3390/pathogens14010058

**Published:** 2025-01-11

**Authors:** Tayane B. S. Magalhães, Amanda de O. Viana, Thiago B. F. Semedo, Juliane S. Saldanha, Nicole A. dos Reis, Nathalia de A. Pereira, Rachel V. P. de Barros, Hannah R. Miranda, Gabriella C. Almeida, Desyrée Y. S. R. Ozaki, Giovana S. Caleiro, Gustavo O. Fenner, Fernanda P. Vizu, Theo Kraiser, Thais P. Carvalho, Luciano M. Thomazelli, Erick G. Dorlass, Clarice W. Arns, Helena L. Ferreira, Erika Hingst-Zaher, Rogério Vieira Rossi, Guilherme S. T. Garbino, Edison L. Durigon, Jansen de Araujo, Daniel M. de Aguiar

**Affiliations:** 1 Laboratório de Virologia e Rickettsioses, Faculdade de Medicina Veterinária, Universidade Federal de Mato Grosso, Fernando Correa da Costa, 2367, Cuiabá 78060-900, Brazil; tayane.bruna16@hotmail.com (T.B.S.M.); thiagosemedo@gmail.com (T.B.F.S.); jusaldanhasilva@gmail.com (J.S.S.); nathaliaassis89@gmail.com (N.d.A.P.); rachelvieirab@gmail.com (R.V.P.d.B.); hannah.rodrigues@hotmail.com (H.R.M.); gabriella.c.almeida@gmail.com (G.C.A.); 2 Laboratório de Virologia Clínica e Molecular, Instituto de Ciências Biomédicas, Universidade de São Paulo, São Paulo 05508-000, Brazil; viana@usp.br (A.d.O.V.); lucmt@usp.br (L.M.T.); erick.dorlass@einstein.br (E.G.D.); eldurigo@usp.br (E.L.D.); 3 Centro de Investigação em Biodiversidade e Recursos Genéticos (CIBIO), InBIO Laboratório Associado, Universidade do Porto, 4099-002 Porto, Portugal; 4 Program in Genomics, Biodiversity and Land Planning (BIOPOLIS), CIBIO, Universidade do Porto, 4099-002 Porto, Portugal; 5 Departamento de Biologia, Faculdade de Ciências, Universidade do Porto, 4099-002 Porto, Portugal; 6 Laboratório de Pesquisa em Vírus Emergentes, Instituto de Ciências Biomédicas, Universidade de São Paulo, São Paulo 05508-000, Brazil; nicole.reis.esib@esib.butantan.gov.br (N.A.d.R.); desyumi.walker@gmail.com (D.Y.S.R.O.); giovanacaleiro@gmail.com (G.S.C.); fernandap.vizu@icb.usp.br (F.P.V.); theokraiser@gmail.com (T.K.); thaispailocarvalho@usp.br (T.P.C.); 7 Museu Biológico, Instituto Butantan, São Paulo 05585-000, Brazil; erika.zaher@butantan.gov.br; 8 Varsomics, Hospital Israelita Albert Eisntein, São Paulo 05652-900, Brazil; 9 Departamento de Genética, Evolução, Microbiologia e Imunologia, Instituto de Biociências, Universidade Estadual de Campinas, Campinas 13083-862, Brazil; clarns@gmail.com; 10 Faculdade de Zootecnia e Engenharia de Alimentos, Universidade de São Paulo, Pirassununga 13635-900, Brazil; hlage@usp.br; 11 Laboratorio de Mastozoologia, Instituto de Biociencias, Universidade Federal de Mato Grosso, Cuiabá 78060-900, Brazil; rogerrossi@gmail.com; 12 Museu de Zoologia João Moojen, Departamento de Biologia Animal, Universidade Federal de Viçosa, Viçosa 36570-900, Brazil; guilherme.garbino@ufv.br

**Keywords:** Orthocoronavirus, Chiroptera, molecular detection, Pancoronavirus RT-PCR, western Brazil

## Abstract

Coronaviruses (CoV) infect a wide variety of hosts, causing epidemics in humans, birds, and mammals over the years. Bats (order Chiroptera) are one of the natural hosts of the Coronaviridae family. They represent 40% of the total number of mammal species in the Pantanal, a biodiversity hotspot in South America. Given the recent SARS-CoV-2 pandemic, we investigated the presence of CoV in bats captured in the Brazilian Pantanal. Oral and rectal swabs collected in 2021 from 419 bats were analyzed using Pancoronavirus-nested PCR targeting the RNA-dependent RNA-polymerase (RdRp) gene. Orthocoronavirinae was detected in 16.7% (70/419) of the bats; nine samples were sequenced, confirming that *Carollia perspicillata* (4), *Phyllostomus hastatus* (2), *Desmodus rotundus* (1), *Molossus rufus* (1), and *Myotis* cf. *nigricans* (1) collected in buildings formally used by humans were infected by *Alphacoronavirus* genera. This is the first description of *Alphacoronavirus* in bats from the Pantanal. As they are natural reservoirs of CoVs, constant monitoring of bats is important to comprehend the epidemiology of emerging viruses, especially in the Pantanal biome.

## 1. Introduction

In recent decades, there has been a significant increase in the occurrence of emerging viral diseases of a zoonotic nature. Due to anthropogenic changes in the environment, the habitat of different species of wild animals is decreasing, affecting their ecological niches and narrowing the contact between these animals and humans. Consequently, these events may reflect in the emergence or re-emergence of zoonotic diseases [1,2].

Coronaviruses (CoVs) are among the major emerging viral pathogens and have been responsible for the largest pandemics in humans and animals over the years. They belong to the Orthocoronavirinae subfamily, which is composed of the *Alphacoronavirus* and *Betacoronavirus* genera responsible for causing infections in mammals and the *Deltacoronavirus* and *Gamacoronavirus* primarily infecting birds [3,4]. High rates of genetic plasticity present in CoVs allow them to adapt rapidly to different hosts [5,6], causing infections in a wide variety of mammals, for example, and the occurrence of spillovers [7].

According to the International Committee on Taxonomy of viruses (ICTV 2021), the *Alphacoronavirus* genus is divided into 15 subgenera; however, the most recent classification considers that CoVs detected in American bats have been classified into seven clades (A to G) [8,9]. Bats (order Chiroptera) are natural reservoirs of approximately 60% of *Alpha* and *Betacoronaviruses*, including SARS-CoV and MERS-CoV, which are *Betacoronaviruses* pathogenic to humans. Furthermore, except for Antarctica, bats are widely distributed on all continents. Global bat diversity is estimated to comprise 22% of all mammal species in the world, with more than 1400 different species [4,10].

Emerging zoonoses in tropical regions with rich biodiversity have increased in recent decades due to anthropogenic actions in natural habitats and the great availability of vectors and reservoir hosts [2]. Bat-CoV has been reported in different biomes in Brazil. *Alphacoronavirus* in bats has been detected in forested areas of the Atlantic Forest and other locations in the states of São Paulo and Bahia, for example [11,12,13,14]. *Betacoronavirus*, however, has only been detected in bats from forested areas of the Atlantic Forest [14]. The state of Mato Grosso, located in the central–western region of Brazil and central South America, encompasses three biomes of great diversity: the Pantanal, Cerrado (Brazilian savannah), and the Amazon. The order Chiroptera represents 40% of the mammal species existing in the Pantanal, and among the 186 bat species recorded in Brazil, 115 occur in the Cerrado and 52 occur in the Pantanal biomes [15,16]. For the state of Mato Grosso, 99 species have been recorded. CoV infection in bats has never been studied in the Mato Grosso biomes, especially in the Pantanal, the World’s largest wetland located in the middle of the South American continent.

Considering the vast diversity of Chiroptera in Mato Grosso State and the absence of Bat-CoV detection in the region, as well as the recent SARS-CoV-2 pandemic, our objective was to detect and analyze the presence of CoV in bats captured in the Pantanal wetland and the surrounding Cerrado biomes.

## 2. Materials and Methods

### 2.1. Bat Capture and Sample Collection

**Figure 1:**
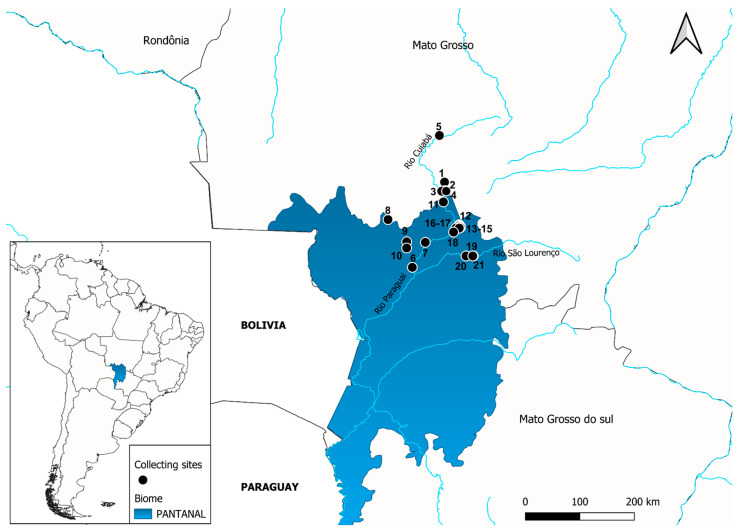
The geographic location of bat collection sites in the Pantanal and Cerrado biomes of Mato Grosso State, Brazil. The numbering of sites follows the sequence of locations presented in Table 1.

We conducted 18 field campaigns during 2021 to collect bats in forested habitats, anthropized areas, bays, peri-urban, and urban areas (abandoned buildings) of the Pantanal and Cerrado biomes, covering the municipalities of Cuiabá, Nobres, Poconé, and Santo Antônio do Leverger (Table 1; Figure 1).

Capture methods included the use of mist nets and active search with the aid of a hand net. Two oral and two rectal swabs were collected from each bat and stored in viral transport medium (VTM) in three vials, one mixed vial containing one swab from each mucosa and two individual vials. The vials were stored in liquid nitrogen containers. Some bats were euthanized for organ collection and deposited in the mammal collection of Universidade Federal de Mato Grosso (UFMT). Bats were identified at the species level according to morphological characteristics following taxonomic keys [17].

The capture of bats and the collection of biological materials were authorized by the Ethics Committee on the Use of Animals of UFMT (CEUA; 23108.085496/2020-32) and the Chico Mendes Institute for Biodiversity Conservation, Brazilian Ministry of the Environment (SisBio/ICMbio: 76825-1; 77787).

### 2.2. Genetic Extraction and RT-Nested-PCR for Orthocoronavirinae Detection

Oral and rectal swabs from each bat were grouped into micro tubes and then subjected to extraction of genomic material in an automatic DNA/RNA extractor, MAGMAX™ FLEX (Applied Biosystems, Waltham, MA, USA), using the MagMax Core Nucleic Acid Isolation Kit (Thermo Fisher Scientific, Waltham, MA, USA) according to the manufacturer’s recommendations. After extraction, the genetic material was quantified in a NanoDrop™ (Thermo Fisher Scientific, Seattle, WA, USA) and stored at −80 °C until analysis.

The extraction products were subjected to RT-nestedPCR reaction for molecular detection of the *Orthocoronavirinae* subfamily, as described by Chu et al. [18], using the RNA-dependent RNA-polymerase (RdRp) gene as the target region (Table 2). The SuperScript III One-Step RT-PCR System with Platinum *Taq* DNA Polymerase (Invitrogen Corporation, Waltham, MA, USA) was used for the amplification reaction following the manufacturer’s protocol with adaptations according to Chu et al. [18]. The amplification reaction was performed in a SimpliAmp Thermal Cycler machine (Applied Biosystems, Foster, CA, USA). PCR products were loaded on 1.5% agarose gel stained with GelRed™ Nucleic Acid Gel Stain (Biotium, Fremont, CA, USA) and visualized on a ChemiDoc XRS system (Bio-Rad, Hercules, CA, USA).

### 2.3. Nucleotide Sequencing and Phylogenetic Analysis

Ten amplicons of the expected size were purified using the Reliaprep DNA Clean-up and concentration system kit (Promega^®^, Madison, WI, USA) and prepared for sequencing with the BigDye™ Kit (Applied Biosystems, Foster, CA, USA). An ABI-PRISM 3500 Genetic Analyzer (Applied Biosystems, Foster, CA, USA) was used for sequencing procedures with the same primers used for PCR. The sequences were assembled using Geneious Prime^®^ software (version 2019.1.1, Biomatters, New Zealand, OC, USA) and compared with sequences previously deposited in the GenBank^®^ database.

For the RdRp nucleotide analyses, a dataset comprising 41 available GenBank sequences representing the Alpha and Beta genera was used, and six sequences were obtained in this study. Sequence alignment was performed with MAFFT [19] and edited with Aliview [20]. Phylogenetic trees estimated using Maximum Likelihood were constructed using IQ-tree2 [21] with 1000 replications on the UltraFast bootstrap using the TIM3 + F + I + G4 substitution model chosen with ModelFinder [22]. For tree editing, we used IToL. The obtained sequences for the bat *Alphacoronaviruses* were submitted to GenBank and are available under the accession numbers PQ493292 to PQ493297.

## 3. Results

A total of 419 bats were captured in different habitats in the Pantanal and Cerrado. From the total bat specimens captured, 202 were identified in the field and released after collecting the samples. Another 217 specimens were euthanized for identification and deposited in the mammal collection of UFMT. After euthanasia, swabs, organs, and tissues were collected and then the carcasses were stored in 70% alcohol.

The highest richness was recorded in the Phyllostomidae family, with 14 species, followed by the Molossidae family with five species, Vespertilionidae with four species, Noctilionidae with two species, and Emballonuridae and Mormoopidae with only one species for each family. The greatest abundance of individuals was in the Phyllostomidae family, with 332 specimens captured. The most abundant species was *Glossophaga soricina*, which accounted for 110 captured specimens. Table 3 presents the list of bat species included in this report.

Seventy out of the 419 samples (16.7%) showed amplification of 440 base pair (bp) products consistent with CoVs’ RT-PCR RdRp gene. These samples covered 15 species (Table 4) collected in three municipalities: forest areas, bays, farms/ranches, and urban and peri-urban areas. Of the positive bat species, *Carollia perspicillata* (19 individuals) prevailed, followed by *G. soricina* (18 individuals), and *Phyllostomus hastatus* (8 individuals). The municipality of Santo Antônio do Leverger presented the highest number of positive samples (Table 4).

Partial sequences of the RdRp gene were obtained from six sequences with 330 bp, and one sequence with 160 bp, 142 bp, and 129 bp each. The longest sequences were from one *C. perspicillata* (identification code: SP04) sampled at Rio Claro, Poconé municipality, and three others (SL61, SL64, and SL74) sampled in the Tamandua region, Santo Antonio do Leverger municipality; one *Desmodus rotundus* (JS199) sampled in the Rio Claro region, Poconé; and one *P. hastatus* (JS185) sampled at Recanto AME+, Santo Antonio do Leverger. The similarity values of these sequences ranged from 75 to 100% when compared with one another. When analyzed against CoV sequences from different locations and bats deposited in GenBank, they showed nucleotide identities varying from 92% to 98% with other bat *Alphacoronaviruses*. The sequences from *C. perspicillata* showed similarities between 96 and 98% compared to isolates detected in *C. perspicillata* and *P. lineatus* from the state of Ceará, the northeastern region of Brazil. The sequence of *D. rotundus* had 98% identity with isolates detected in *D. rotundus* captured in São Paulo (Brazil) and Argentina. A nucleotide identity of 97% was observed between the CoV sequence detected in *P. hastatus* and isolates detected in *C. perspicillata* and *P. linneatus* captured in Ceará and *A. planirostris* from São Paulo State, Brazil. Given that these nine sequences represented 12.85% of the RT-PCR products, we considered the other detected samples as suspicious.

The remaining minor sequences were identified in samples of *G. soricina* (AFT7), *Myotis* cf. *nigricans* (JS204), and *P. hastatus* (JS206). These last nucleotides sequences, although smaller, presented identities of 98% (AFT7) with bat *Alphacoronavirus* of the *C. perspicillata* isolate and 96% (JS204; JS206) for bat *Alphacoronavirus* detected in *Myotis riparius* and *Myotis* sp.

**Figure 2:**
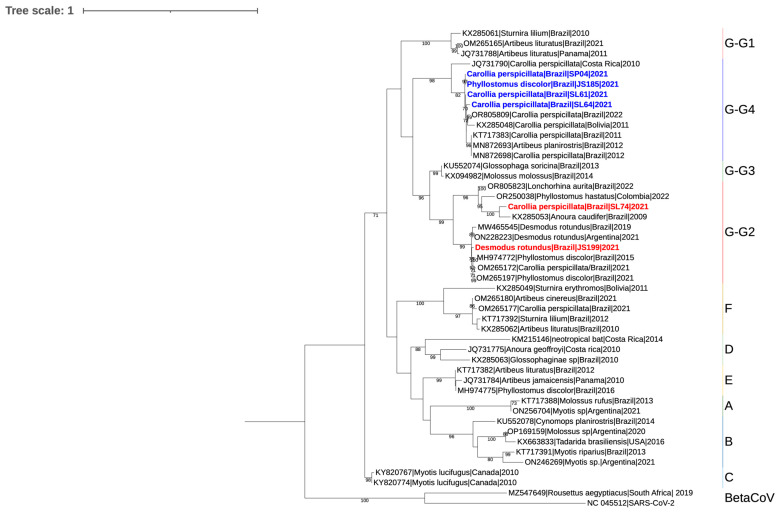
Phylogenetic tree of the Coronavirus RdRp genomic region using the Maximum Likelihood algorithm constructed using IQ-tree2 with 1000 replications on the UltraFast bootstrap using TIM3 + F + I + G4 substitution model, with a total of 330 nucleotide positions in the final data set, based on six sequences from this study (blue and red) and 41 representative sequences of *Alphacoronavirus* and *Betacoronavirus* available in GenBank. The nodes show bootstrap values higher than 70. The tree shows the subgenera classified into A to G, and subgenera G is separated by subclades. The samples in this study belong to G2 and G4. Sequences from the genus *Betacoronavirus* (*Roussettus aegyptacus*) from South Africa were used as an outgroup to root the phylogenetic tree.

The phylogenetic analysis conducted on the RdRp gene sequences of Bat-CoV demonstrated that three sequences detected in *C. perspicillata* (SL61, SL64, and SP04) and one in *P. hastatus* (JS185) grouped in a subclade of the subgenera G (G4) with a bootstrap value of 98 with other sequences detected in *C. perspicillata* and *P. lineatus* from the northeastern region of Brazil and Costa Rica and *Artibeus planirostris* from the state of São Paulo, Brazil. The sequence detected in *D. rotundus* (JS199) and *C. perspicillata* (SL74) nested with another subclade of subgenera G (G2) supported by the bootstrap value of 99, separated into two additional branches the first of which (bootstrap value of 99) includes several sequences from *D. rotundus* and *P. discolor* and another (bootstrap value of 96) that includes Bat-CoV sequences detected in *Anoura caudifer, P. hastatus,* and *Lonchorhina aurita* from different regions of Brazil, Argentina, and Colombia (Figure 2).

## 4. Discussion

This study is the first to detect Orthocoronavirus in bats from the Pantanal in Mato Grosso State. Given the high biodiversity of mammals in this biome and especially the presence of several families of chiropterans, a significant number of suspicious bats were detected (16.7% of the captured individuals). Our study was limited to sequencing nine samples, but this was sufficient to demonstrate for the first time the involvement of the *Alphacoronavirus* genus in Pantanal chiropteran fauna. We consider them as suspicious because they presented an amplification of 440 base pairs, which was expected according to the protocol described by Chu et al. [18]. However, we were able to confirm only nine positive samples among the amplicons obtained. From all the amplicons detected (n = 70), we chose ten with the best concentration rate for sequencing, and nine were confirmed. Furthermore, only from six samples was it possible to obtain fragments of 330 bp. These results demonstrate a high number of amplicons with insufficient quantity for sequencing and should be considered a limitation of the present study. Despite this, the protocol of Chu et al. [18] used in our study has been adopted in different Orthocoronavirus surveys and monitoring in Brazil, mainly in different states within the Atlantic Forest or Amazonia biomes [6,9,11,12,13,14], making our results relevant. Other investigations have also used protocols targeting amplification in the RdRp region (Pancoronavirus RT-PCR) but with different primers hybridizing different regions of the gene with some overlap [7,23,24,25], which emphasize, however, the relevance of this target that is highly conserved among the different CoVs and provides sequences of sufficient length for phylogenetic analysis [25]. Thus, we emphasize that our procedures sought to follow former studies, minimizing deleterious methodological influence and allowing for the comparison of results with other studies.

The high biodiversity of the Pantanal fauna was reflected in the richness of the families and genera of bats. Capture of common frugivorous bats predominated, in addition to insectivorous species. The expressive capture of these species reinforces the greater concentration of these animals in areas of forest edges and fragments, water bodies, and open areas, where there is a greater abundance of food, especially in ecologically preserved regions [26,27]. In addition, we also highlight the capture of bats in urbanized environments, such as abandoned houses and the backyards of other inhabited homes.

Although this is the first detection of *Alphacoronavirus* in bats from the Pantanal, in addition to the Brazilian Atlantic Forest and the Amazon, CoV-infected bats have been identified in 11 countries on the American continent [28,29]. Areas with high biological diversity such as the Pantanal have been sought after for tourist activities, which is why some areas have preserved natural environments; other areas, however, have undergone major anthropogenic transformations and offer chances of becoming foci of future epidemics [30]. Major environmental disasters such as those that occurred in 2020 with the wildfires in the Pantanal also harm biodiversity, favoring the spread of pathogens. For example, it is estimated that 40,000 km^2^ of habitat were lost during the wildfires in the Pantanal, with the death of 17 million vertebrates, not counting bats, causing great environmental instability in the region [31,32,33]. In the present study, we highlight that all detections of Alphacoronavirus in bats were from specimens captured in the backyards of abandoned buildings, near homes, demonstrating that despite the size of the territory investigated and several preserved forest areas in the state of Mato Grosso, these animals dwell in environments close to humans, bringing these viral agents into proximity. The deterioration of habitat quality, increased population density in residual fragments with the reduction in food availability, and climate change, for example, result in weaker individuals susceptible to infections [31,33,34].

The municipality with the highest number of positive bats (84.3%) was Santo Antônio do Leverger, also the one with the most captures. Of the 11 capture locations, it was possible to detect positive bats in five (45.5%), with emphasis on the species *C. perspicillata*, which presented a positive frequency of 23.7%, *G. soricina* with 16%, and *N. albiventris* and *Myotis.* cf. *nigricans*, both with 25% each. The frequency of CoVs in *C. perspicillata* in the present study exceeded the detection rate (3%) described in the review of Hernández-Aguilar et al. [28], who analyzed results from 25 studies on *Alphacoronaviruses* in bats in the Americas. Infection in this bat species has been described in several American countries, such as Costa Rica, Bolivia, Brazil, Ecuador, Mexico, Panama, Peru, and Trinidad and Tobago. CoV sequences from *C. perspicillata* (SL61, SL64, SP04) and *P. hastatus* (JS185) bats were positioned in a monophyletic clade (bootstrap of 98) composed of samples belonging to the subgenus G4 that was detected predominantly in the same bat species from Costa Rica, Brazil, and Bolivia, in addition to *A. planirostris* and *P. lineatus* from Brazil. Of these four, bats from G4, SL61, and SL64 were captured in the Tamandua region, while one bat (JS185) was captured at the Recanto AME+ site, both in the municipality of Santo Antonio do Leverger. In contrast, only Bat SP04 was captured in Poconé (Rio Claro region). These results demonstrate that the subgenus G4 is distributed in different locations of the Pantanal-infecting bats of the Phyllostomidae family.

The fourth sequence (SL74) detected in *C. perspicillata* formed a monophyletic group in the clade of subgenus G2 (bootstrap of 96), more specifically paired in a subclade composed of CoV detected in *A. caudifer* from Brazil (bootstrap support of 100), and close to sequences detected in *L. aurita* from Brazil and *P. hastatus* from Colombia. *Anoura caudifer* are important in seed dispersal and act as forest pollinators. It is worth highlighting the habitation of these species that form colonies in caves, abandoned houses, tunnels or water pipes, bridges, and tree hollows, among other environments that may facilitate the sharing of places by different species, facilitating the dispersion of a certain group of *Alphacoronaviruses* among different species of bats. Of the specimens mentioned, only *P. hastatus* was captured in this study (JS185); however, as per the paragraph above, this sequence was classified in clade G4.

Another monophyletic group (bootstrap of 99) of this same clade (G2) contained our sequence detected in *D. rotundus* (JS199), which was close to other specimens of the same species from Brazil and Argentina. This positioning corroborates the findings described by Arteaga et al. [9] in Argentina; as observed in our phylogenetic tree, the sequences detected in *D. rotundus* in their study also presented a branch close to a group of *Alphacoronaviruses* detected in the *Phyllostomus* genus. These two species inhabit shelters represented by abandoned houses or caves, forming large colonies, which may eventually co-inhabit and facilitate the sharing of *Alphacoronavirus* of the subgenus G2.

Interestingly, the G2 clade presented herein and by Arteaga et al. [9] contains sequences (such as OM265189 and OM265196) classified as *Amalacovirus* by Bueno et al. [13]. *Amalacovirus* refers to a subgenus within the *Alphacoronavirus* genus, which falls under the family Coronaviridae. These viruses primarily infect bats and are part of the Orthocoronavirinae subfamily. Research into Amalacoviruses has been limited and focused mostly on a narrow range of bat species, like *D. rotundus*, known as the common vampire bat. The initial assumption was that only hematophagous (blood-feeding) bats carried these viruses, but recent international studies have shown a more complex reality. Evidence now in our findings indicates that Amalacoviruses are found in various species of frugivorous bats (fruit-eating), suggesting a broader host range that is not strictly linked to feeding habits but rather influenced by bat roosting behavior and environmental factors. Similar results were found in previous reports in Brazil [13]. Moreover, most existing data have been derived from studies outside Brazil, emphasizing a lack of localized research in regions like South America, where new observations are challenging these earlier notions about host specificity and ecological associations.

## 5. Conclusions

This is the first description of *Alphacoronavirus* in bats from the Pantanal, the world's largest wetland, located in Mato Grosso State, Brazil. At least two *Alphacoronavirus* subgenera are present in this region, and they seem to be closely related to specific host species. Since the Pantanal is a region with high biodiversity, it is extremely important that there are long-term monitoring programs for this viral variability. As bats are natural reservoirs of CoVs, constant monitoring of these animals is important to comprehend the epidemiology of emerging viruses, especially in this enigmatic and poorly known biome.

## Figures and Tables

**Table 1:** Geographic coordinates of bat collection sites and their municipalities and localities in the Pantanal and Cerrado biomes, Mato Grosso State, Brazil.

Municipality	Location	Geographic Coordinates
Cuiabá	1 Coxipó do ouro	−15°27′25″; −056°03′50″
	2 INPP	−15°36′23″; −056°03′45″
	3 Urban area NDL	−15°36′36″; −056°07′28″
	4 CK Equine medicine	−15°37′25″; −056°02′36″
Nobres	5 Pé-de-serra ranch	−14°37′48″; −056°09′27″
Poconé	6 São João ranch	−16°56′40″; −056°38′15″
	7 Porto Cercado	−16°30′43″; −056°24′57″
	8 São Pedro ranch	−16°06′55″; −057°03′47″
	9 Pouso Alegre	−16°30′11″; −056°44′44″
	10 Rio Claro	−16°37′07″; −056°44′13″
Santo Antônio do Leverger	11 Recanto AME+	−15°48′22″; −056°04′53″
	12 Mimoso	−16°11′42″; −055°48′25″
	13 Mimoso	−16°12′40″; −055°48′26″
	14 Mimoso	−16°14′15″; −055°46′45″
	15 Memorial do Rondon	−16°15′14″; −055°47′12″
	16 Baía de Chacororé	−16°15′49″; −055°51′32″
	17 Mimoso	−16°16′04″, −055°47′46″
	18 Baía de Sinhá Mariana	−16°20′24″; −055°53′57″
	19 Baía São João	−16°44′31″; −055°33′05″
	20 Tamanduá	−16°45′03″; −055°40′36″
	21 São Lourenço	−16°45′16″; −055°34′02″

INPP: Instituto Nacional Pesquisa do Pantanal.

**Table 2:** Nucleotide sequences of the primers used in the molecular detection of the Orthocoronavirinae (CoV) subfamily.

Forward 1	CHU1F: 5′ GGKTGGGAYTAYCCKAARTG 3′
Reverse 1	CHU1R: 5′ TGYTGTSWRCARAAYTCRTG 3′
Forward 2	CHU2F: 5′ GGTTGGGACTATCCTAAGTGTGA 3′
Reverse 2	CHU2R: 5′ CCATCATCAGATAGAATCATCAT 3’

**Table 3:** List of bats captured for the Coronavirus survey according to family, species, and abundance.

Family	Species	Abundance (n°)
Phyllostomidae	*Glossophaga soricina*	110
	*Carollia perspicillata*	80
	*Artibeus planirostris*	33
	*Artibeus obscurus*	25
	*Platyrrhinus lineatus*	22
	*Phyllostomus hastatus*	19
	*Desmodus rotundus*	15
	*Macrophyllum macrophyllum*	14
	*Trachops cirrhosus*	9
	*Lophostoma silvicola*	2
	*Lophostoma brasiliense*	1
	*Sturnira lilium*	1
	*Carollia* sp.	1
Molossidae	*Molossops temminckii*	6
	*Molossus molossus*	5
	*Molossus rufus*	5
	*Cynomops planirostris*	2
	*Molossus* sp.	1
Vespertilionidae	*Myotis* cf. *nigricans*	29
	*Eptesicus* sp.	2
	*Myotis* sp.	1
	*Rhogeessa io*	1
Noctilionidae	*Noctilio albiventris*	24
	*Noctilio leporinus*	1
Emballonuridae	*Rhynchonycteris naso*	6
Mormoopidae	*Pteronotus* sp.	4

**Table 4:** Number of positive bats for Orthocoronavirus and their prevalence, according to family, species, frequency, and collection site.

Family	Species (%)	Locality	Number
Phyllostomidae	*Artibeus planirostris* (3%)	Coxipó do ouro	1
	*Carollia perspicillata* (23.7%)	Baía de São João	9
		Baía de Sinhá Mariana	1
		Tamanduá	5
		Mimoso	3
		Rio Claro	1
	*Desmodus rotundus* (6.6%)	Rio Claro	1
	*Glossophaga soricina* (16%)	Baia de São João	6
		Coxipó do ouro	2
		Mimoso	8
		Pouso Alegre	2
	*Phyllostomus hastatus* (42.1%)	Mimoso	2
		Recanto AME+	1
		Baía de São João	5
	*Platyrrhinus lineatus* (7.7%)	Baía de Sinhá Mariana	1
Molossidae	*Cynomops planirostris* (100%)	Baía de Sinhá Mariana	2
	*Molossus molossus* (20%)	Baía de Sinhá Mariana	1
	*Molossus rufus* (20%)	Recanto AME+	1
	*Molossus* sp. (20%)	Coxipó do Ouro	1
Mormoopidae	*Pteronotus* sp. (75%)	Coxipó do Ouro	3
Noctilionidae	*Noctilio albiventris* (25%)	Baía de Sinhá Mariana Mimoso	5 1
Vespertilionidae	*Myotis* cf. *nigricans* (100%)	Baía de Sinhá Mariana	1
		Baía de São João	6
		Mimoso	1

## Data Availability

The sequences are available under GenBank accession numbers PQ493292 to PQ493297.

## References

[1] Tazerji S.S., Nardini R., Safdar M., Shehata A.A., Duarte P.M. (2022). An Overview of Anthropogenic Actions as Drivers for Emerging and Re-Emerging Zoonotic Diseases. Pathogens.

[2] Ellwanger J.H., Fearnside P.M., Ziliotto M., Valverde-Villegas J.M., Veiga A.B.G.D., Vieira G.F., Bach E., Cardoso J.C., Müller N.F.D., Lopes G. (2022). Synthesizing the Connections between Environmental Disturbances and Zoonotic Spillover. Acad. Bras. Cienc..

[3] Cruz-Pulido D., Ouma W.Z., Kenney S.P. (2022). Differing Coronavirus Genres Alter Shared Host Signaling Pathways upon Viral Infection. Sci. Rep..

[4] Weber M.N., da Silva M.S. (2023). Corona- and Paramyxoviruses in Bats from Brazil: A Matter of Concern?. Animals.

[5] Sikkema R.S., Koopmans M.P.G. (2021). Preparing for Emerging Zoonotic Viruses. Encycl. Virol..

[6] Barbosa C.M., Durigon E.L., Thomazelli L.M., Ometto T., Marcatti R., Nardi M.S., de Aguiar D.M., Pinho J.B., Petry M.V., Neto I.S. (2019). Divergent Coronaviruses Detected in Wild Birds in Brazil, Including a Central Park in São Paulo. Braz. J. Microbiol..

[7] Marchenko V., Danilenko A., Kolosova N., Bragina M., Molchanova M., Bulanovich Y., Gorodov V., Leonov S., Gudymo A., Onkhonova G. (2022). Diversity of Gammacoronaviruses and Deltacoronaviruses in Wild Birds and Poultry in Russia. Sci. Rep..

[8] Caraballo D.A. (2022). Cross-Species Transmission of Bat Coronaviruses in the Americas: Contrasting Patterns between Alphacoronavirus and Betacoronavirus. Microbiol. Spectr..

[9] Arteaga F.L., Miragaya M., Molina N., Mondino M.A., Bracamonte J.C., Capitelli G.M., Mundo S., Bratanich A.C. (2022). Circulation of Coronavirus in Bats from Northern and Central Argentina: Preliminary Study. Int. J. Infect. Dis..

[10] Letko M., Seifert S.N., Olival K.J., Plowright R.K., Munster V.J. (2020). Bat-Borne Virus Diversity, Spillover and Emergence. Nat. Rev. Microbiol..

[11] Asano K.M., Hora A.S., Scheffer K.C., Fahl W.O., Iamamoto K., Mori E., Brandão P.E. (2016). Alphacoronavirus in Urban Molossidae and Phyllostomidae Bats, Brazil. Virol. J..

[12] Bittar C., Machado R.R.G., Comelis M.T., Bueno L.M., Beguelini M.R., Morielle-Versute E., Nogueira M.L., Rahal P. (2020). Alphacoronavirus Detection in Lungs, Liver, and Intestines of Bats from Brazil. Microb. Ecol..

[13] Bueno L.M., Rizotto L.S., de Oliveira Viana A., Silva L.M.N., de Moraes M.V.D.S., Benassi J.C., Scagion G.P., Dorlass E.G., Lopes B.L.T., Cunha I.N. (2022). High Genetic Diversity of Alphacoronaviruses in Bat Species (Mammalia: Chiroptera) from the Atlantic Forest in Brazil. Transbound. Emerg. Dis..

[14] Góes L.G.B., de Almeida Campos A.C., de Carvalho C., Ambar G., Queiroz L.H., Cruz-Neto A.P., Munir M., Durigon E.L. (2016). Genetic Diversity of Bats Coronaviruses in the Atlantic Forest Hotspot Biome, Brazil. Infect. Genet. Evol..

[15] Abreu E.F., Casali D., Costa-Araújo R., Garbino G.S.T., Libardi G.S., Loretto D., Loss A.C., Marmontel M., Moras L.M., Nascimento M.C. (2023). Lista de Mamíferos do Brasil.

[16] Garbino G.S.T., Cláudio V.C., Gregorin R., Lima I.P., Loureiro L.O., Moras L.M., Moratelli R., do Nascimento M.C., Nogueira M.R., Novaes R.L.M. (2024). Updated Checklist of Bats (Mammalia: Chiroptera) from Brazil. Zoologia.

[17] Reis N.R.D., Peracchi A.L., Pedro W.A., Lima I.P. (2007). De Morcegos Do Brasil.

[18] Chu D.K.W., Leung C.Y.H., Gilbert M., Joyner P.H., Ng E.M., Tse T.M., Guan Y., Peiris J.S.M., Poon L.L.M. (2011). Avian Coronavirus in Wild Aquatic Birds. J. Virol..

[19] Katoh K., Standley D.M. (2013). MAFFT Multiple Sequence Alignment Software Version 7: Improvements in Performance and Usability. Mol. Biol. Evol..

[20] Larsson A. (2014). AliView: A Fast and Lightweight Alignment Viewer and Editor for Large Datasets. Bioinformatics.

[21] Minh B.Q., Schmidt H.A., Chernomor O., Schrempf D., Woodhams M.D., von Haeseler A., Lanfear R. (2020). IQ-TREE 2: New Models and Efficient Methods for Phylogenetic Inference in the Genomic Era. Mol. Biol. Evol..

[22] Kalyaanamoorthy S., Minh B.Q., Wong T.K.F., von Haeseler A., Jermiin L.S. (2017). ModelFinder: Fast Model Selection for Accurate Phylogenetic Estimates. Nat. Methods.

[23] Simas P.V.M., de Souza Barnabé A.C., Durães-Carvalho R., de Lima Neto D.F., Caserta L.C., Artacho L., Jacomassa F.A.F., Martini M.C., dos Santos M.M.A.B., Felippe P.A.N. (2015). Bat Coronavirus in Brazil Related to Appalachian Ridge and Porcine Epidemic Diarrhea Viruses. Emerg. Infect. Dis..

[24] Góes L.G.B., Ruvalcaba S.G., Campos A.A., Queiroz L.H., de Carvalho C., Jerez J.A., Durigon E.L., Dávalos L.I.I., Dominguez S.R. (2013). Novel Bat Coronaviruses, Brazil and Mexico. Emerg. Infect. Dis..

[25] Wacharapluesadee S., Thippamom N., Hirunpatrawong P., Rattanatumhi K., Sterling S.L., Khunnawutmanotham W., Noradechanon K., Maneeorn P., Buathong R., Paitoonpong L. (2024). Comparative Performance in the Detection of Four Coronavirus Genera from Human, Animal, and Environmental Specimens. Viruses.

[26] Arzi Y., Segoli M., Schäckermann J., Korine C. (2023). Providing Water Sources to Insectivorous Bats for Conservation Biological Control in Arid Date Plantations. Biol. Control.

[27] de Souza Laurindo R., Vizentin-Bugoni J., Tavares D.C., Mancini M.C.S., de Macêdo Mello R., Gregorin R. (2020). Drivers of Bat Roles in Neotropical Seed Dispersal Networks: Abundance Is More Important than Functional Traits. Oecologia.

[28] Hernández-Aguilar I., Lorenzo C., Santos-Moreno A., Naranjo E.J., Navarrete-Gutiérrez D. (2021). Coronaviruses in Bats: A Review for the Americas. Viruses.

[29] Ruiz-Aravena M., McKee C., Gamble A., Lunn T., Morris A., Snedden C.E., Yinda C.K., Port J.R., Buchholz D.W., Yeo Y.Y. (2021). Ecology, evolution and spillover of coronaviruses from bats. Nat. Rev. Microbiol..

[30] Marques J.F., Alves M.B., Silveira C.F., Amaral e Silva A., Silva T.A., dos Santos V.J., Calijuri M.L. (2021). Fires Dynamics in the Pantanal: Impacts of Anthropogenic Activities and Climate Change. J. Environ. Manag..

[31] Tomas W.M., Berlinck C.N., Chiaravalloti R.M., Faggioni G.P., Strüssmann C., Libonati R., Abrahão C.R., do Valle Alvarenga G., de Faria Bacellar A.E., de Queiroz Batista F.R. (2021). Distance Sampling Surveys Reveal 17 Million Vertebrates Directly Killed by the 2020’s Wildfires in the Pantanal, Brazil. Sci. Rep..

[32] Libonati R., DaCamara C.C., Peres L.F., de Carvalho L.A.S., Garcia L.C. (2020). Rescue Brazil’s Burning Pantanal Wetlands. Nature.

[33] Eby P., Peel A.J., Hoegh A., Madden W., Giles J.R., Hudson P.J., Plowright R.K. (2023). Pathogen Spillover Driven by Rapid Changes in Bat Ecology. Nature.

[34] Festa F., Ancillotto L., Santini L., Pacifici M., Rocha R., Toshkova N., Amorim F., Benítez-López A., Domer A., Hamidović D. (2023). Bat Responses to Climate Change: A Systematic Review. Biol. Rev..

